# Low-Molecular Weight Protamine Overcomes Chondroitin Sulfate Inhibition of Neural Regeneration

**DOI:** 10.3389/fcell.2022.865275

**Published:** 2022-04-25

**Authors:** Natalia Kulesskaya, Ekaterina Mugantseva, Rimante Minkeviciene, Natalia Acosta, Ari Rouhiainen, Juha Kuja-Panula, Mikhail Kislin, Sami Piirainen, Mikhail Paveliev, Heikki Rauvala

**Affiliations:** Neuroscience Center, Helsinki Institute of Life Science, University of Helsinki, Helsinki, Finland

**Keywords:** protamine, low-molecular weight protamine, chondroitin sulfates, spinal cord injury, animal models

## Abstract

Protamine is an arginine-rich peptide that replaces histones in the DNA-protein complex during spermatogenesis. Protamine is clinically used in cardiopulmonary bypass surgery to neutralize the effects of heparin that is required during the treatment. Here we demonstrate that protamine and its 14–22 amino acid long fragments overcome the neurite outgrowth inhibition by chondroitin sulfate proteoglycans (CSPGs) that are generally regarded as major inhibitors of regenerative neurite growth after injuries of the adult central nervous system (CNS). Since the full-length protamine was found to have toxic effects on neuronal cells we used the *in vitro* neurite outgrowth assay to select a protamine fragment that retains the activity to overcome the neurite outgrowth inhibition on CSPG substrate and ended up in the 14 amino acid fragment, low-molecular weight protamine (LMWP). In contrast to the full-length protamine, LMWP displays very low or no toxicity in our assays *in vitro* and *in vivo*. We therefore started studies on LMWP as a possible drug lead in treatment of CNS injuries, such as the spinal cord injury (SCI). LMWP mimicks HB-GAM (heparin-binding growth-associated molecule; pleiotrophin) in that it overcomes the CSPG inhibition on neurite outgrowth in primary CNS neurons *in vitro* and inhibits binding of protein tyrosine phosphatase (PTP) sigma, an inhibitory receptor in neurite outgrowth, to its CSPG ligand. Furthermore, the chondroitin sulfate (CS) chains of the cell matrix even enhance the LMWP-induced neurite outgrowth on CSPG substrate. *In vivo* studies using the hemisection and hemicontusion SCI models in mice at the cervical level C5 revealed that LMWP enhances recovery when administered through intracerebroventricular or systemic route. We suggest that LMWP is a promising drug lead to develop therapies for CNS injuries.

## 1 Introduction

Acute injury in the brain and the spinal cord results in massive production of chondroitin sulfate proteoglycans (CSPGs) by activated astrocytes in the posttraumatic scar. CSPGs of the glial scar and their carbohydrate chains have been extensively studied as inhibitors of plasticity and regeneration after trauma. The studies on CSPG inhibition and its role in regulation of CNS plasticity and regeneration have been largely based on the use of chondroitinase ABC that cleaves chondroitin sulfate chains of CSPGs, thereby enhancing plasticity and regeneration (Bradbury et al., 2002; Pizzorusso et al., 2002; Silver and Miller, 2004; Galtrey ad Fawcett, 2007; Garcia-Alias et al., 2009; Sharma et al., 2012). Although a number of strategies have been studied to overcome the glial scar inhibition and to promote recovery from spinal cord injuries (Cregg et al., 2014; Tran et al., 2018), so far no single compound has been developed for medical usage.

Protamine is a small 33 amino acid long protein that takes part in DNA packing during spermatogenesis (Kasinsky et al., 2011). Over 60% of the salmon protamine sequence is comprised of arginines so that it interacts strongly with negatively charged DNA phosphates *in vivo* and potentially with any other negatively charged molecules. Protamine binds the glycosaminoglycan heparin with high affinity and neutralizes its anticoagulant effect. Protamine sulfate is therefore widely used as a drug in cardiopulmonary bypass surgery to stop the effect of heparin (Kaneda et al., 2005; Bromfield et al., 2013). Furthermore, protamine has been used for tens of years in insulin preparations to retard insulin release (World Health Organization, List of essential medicines, 2019). In addition, protamine has been suggested for a range of medical applications including use as a membrane-translocating peptide (Reynolds et al., 2005) and siRNA delivery (Yoo and Mok, 2015; Sokolowska et al., 2016).

Chondroitin sulfates (CS) bare multiple negative charges due to sulfate and carboxyl groups while protamine is a polycation at physiological pH values. Polyion complexation of CS with protamine was demonstrated by attenuated total reflectance Fourier transform infrared spectroscopy (Umerska et al., 2015). Protamine binding to heparin and CSPG (Umerska et al., 2015) suggests that protamine may also mask those CSPG epitopes that inhibit axonal growth. As an FDA-approved drug for human use as anti-heparin, protamine might in addition have potential to overcome the regeneration inhibiting effect of CSPGs and therefore enhance recovery after human CNS injuries. Concerns have been raised of the cell toxicity effects of protamine (Sokolowska et al., 2016) suggesting toxicity-related limitations of its therapeutic potential. Possible approaches for reduction of the protamine toxicity could improve the pharmaceutical value of protamine.

Here we demonstrate that protamine and its fragments block the inhibitory effect of CSPGs on neurite growth in cultured cortical and hippocampal neurons. Protamine is however highly toxic for neuronal cells but the 14 amino acid peptide, known in the literature as the low-molecular weight protamine (LMWP) displaying cell-penetrating properties (He et al., 2014), displays very little or no toxicity for neuronal cells *in vitro* or *in vivo*, and it still retains the activity to block the CSPG inhibition. Furthermore, LMWP enhances recovery in spinal cord hemisection and hemicontusion models in mice.

## 2 Materials and Methods

### 2.1 Protamine and Protamine Fragments

Protamine sulphate from Leo Pharma (Ballerup, Copenhagen, Denmark) was used as the full-length protamine. In some experiments, protamine from Sigma-Aldrich (Saint Louis, MO, United States) was in addition used as the full-length protamine. No differences were observed in the effects of protamine from the different sources. Protamine fragments were synthetized by Caslo ApS (DK-2800, Kongens Lyngby, Denmark). The synthetic low-molecular weight protamine (LMWP; Caslo ApS) with the sequence VSRRRRRRGGRRRR and the fluorescent TAMRA-labelled LMWP (Caslo ApS) were examined in more detail in the current study. The sequence of LMWP (purity 98,4% after HPLC purification) was confirmed by mass spectrometry. The LMWP chloride salt was dissolved in water and stored at −20°C until use.

### 2.2 Cell Culture

The cortex or hippocampus were obtained from 17 days-old Wistar rat embryos and transferred to ice-cold preparation solution. Neurons were further isolated exactly as described earlier in Sahu’s publication (Sahu et al., 2019). Primary rat cortical or hippocampal neurons (E17 Wistar rats) were cultured in 96-well plates at the density of 20000 cells/well in Neurobasal medium supplemented with B27 serum substitute, penicillin, streptomycin and L-glutamate for 2 days *in vitro* (DIV). The plates were precoated with aggrecan (5 ug/ml, 150 µl/well). Protamine or protamine peptides were added to the culture medium at different concentrations. To analyze the effect of protamine or its fragment on neurite outgrowth, the plates were coated with 10 μg/ml of protamine or LMWP. For the experiment with chondroitinase, the aggrecan-coated plates were treated with chondroitinase ABC (Amsbio AMS. E1028, lot CF0029) for 4 h followed by 2 times washing with PBS before cell plating, and the cell culture imaging was carried out after 3 DIV incubation with 4 uM LMWP added to the medium. Neurite outgrowth was imaged with phase contrast microscopy (Olympus IX70 microscope and Retiga 2000R digital camera) using both x10 and x20 objectives. Processes of neurons over the diameter of the neuronal soma were interpreted as neurites. Neurite length was measured with ImageJ software and averaged from 2 to 3 independent experiments with 1–2 technical replicas in each independent experiment.

### 2.3 Cloning and Purification of Protein Tyrosine Phosphataseσ Ectodomains

The N-terminal extracellular part of PTPσ (protein tyrosine phosphatase sigma; PTPRS) that contains the three Ig domains (amino acids 1-321) and mediates CSPG binding (Shen et al., 2009) was cloned using the PTPσ cDNA kindly provided by Dr. John Flanagan (Harvard University). The PTPσ mutant lacking the CS binding site in the Ig1 domain was produced as described (Shen et al., 2009). The recombinants were produced as Fc-tagged proteins in 293T HEK cells and purified on protein A Sepharose.

### 2.4 Solid Phase Binding Assays

The competition assay for LMWP in binding of PTPσ to aggrecan was done in a similar way as earlier (Paveliev et al., 2016). Briefly, after overnight coating with 10 μg/ml aggrecan (150 ul/well) at 4°C 96-well tissue culture plates (Nunc) were washed two times with 0.05% Tween-20 in PBS and were blocked for 1 h at room temperature with 1%BSA and 0.05% Tween-20 in PBS. The recombinant wild type PTPσ ectodomain or the mutated ectodomain lacking CS binding (80 nM) were mixed with different concentrations of LMWP in 1% BSA and 0.05% Tween-20 in PBS, and were incubated in the plates overnight at 4°C. After washing with 0.05% Tween20 (3 times, room temperature), the wells were incubated for 1 h (room temperature) with HRP conjugated goat anti-human FC part of IgG (1:5000, in 1% BSA and 0.05% Tween20 PBS) to detect the bound PTPσ. After another washing (3 times with 0.05% Tween-20 PBS), O-phenylendiamine dihydrochloride (OPD, SIGMA P9187) substrate was added to measure the enzymatic activity of the bound HRP conjugate at 492 nm.

### 2.5 *In Vivo* Studies

#### 2.5.1 Animals

Young adult C57BL6/NHsd females and males (8 weeks old) were obtained from commercial provider and acclimated to the university animal facility for 2 weeks before starting the experiments. Female animals were used for long-term SCI functional studies to avoid fighting in post-surgery period, while both males and females were used for *in vivo* TUNEL apoptosis assay. Animals were group housed by 4–5 animals in IVC cages provided with aspen chip bedding and nesting material, food and water *ad libitum*. Animal facility has controlled 12 h light cycle (light period starts at 6 a.m.), room temperature 21 ± 1°C and relative humidity 50%–60%.

#### 2.5.2 *In Vivo* TUNEL Apoptosis Assay

For TUNEL apoptosis assay, 1.5 μl of protamine (0.1 mg/ml or 1 mg/ml) or LMWP (0.1 mg/ml or 1 mg/ml) was bilaterally injected to the adult C57Bl6 mouse (*n* = 10 total) into motor (ML: 1.5; AP: 2) and somatosensory (ML: 1.5; AP: −1.8) cerebral cortex 500–700 micrometers below the brain surface (4 injection sites per concentration of injected protamine and 6 injections per concentration of injected LMWP). After 3 days the animals were anesthetized with pentobarbital sodium and perfused with PBS and then 4%PFA. After paraffine embedding the brains were cut into 7 µm coronal sections. Staining was performed according to the protocol recommended by the commercial TUNEL assay kit (Dead End Fluorometric TUNEL System, Promega). Sections treated with DNase I were used as positive controls. Vectoshield medium with DAPI (Vector Laboratories) was used to visualize nuclei. Images were collected with Axio Imager M.1 fluorescent microscope (Zeiss), AxioCam HR3 digital camera and x10 0.45 NA objective (Carl Zeiss Microscopy) and analyzed with automatic cell counting using FIJI software (Image J, open-source). The fractions of TUNEL positive cells were calculated and averaged over 5 sections per mouse per injection place.

#### 2.5.3 Distribution of Fluorescent Low-Molecular Weight Protamine After Subcutaneous Administration

The TAMRA-labelled LMWP was obtained from commercial provider Caslo ApS (DK-2800, Kongens Lyngby, Denmark). Sixteen micrograms of TAMRA-LMWPor unconjugated TAMRA (in 200 ul of 0.9% NaCl) was injected subcutaneously to upper back area to healthy adult mice (*n* = 2–3 animals/group). At the end of the experiment (2 h after injection), the animals were perfused with PBS and then with 4% paraformaldehyde (PFA) in PBS. The spinal cord was dissected and further fixed in PFA overnight. One hundred µm-thick longitudinal sections of the cervical spinal cord were cut with vibratome and imaged with Zeiss LSM880 confocal, 20x objective.

#### 2.5.4 Mouse Models of Spinal Cord Injury

##### 2.5.4.1 Schedule of Spinal Cord Injury Studies

**FIGURE 1:**
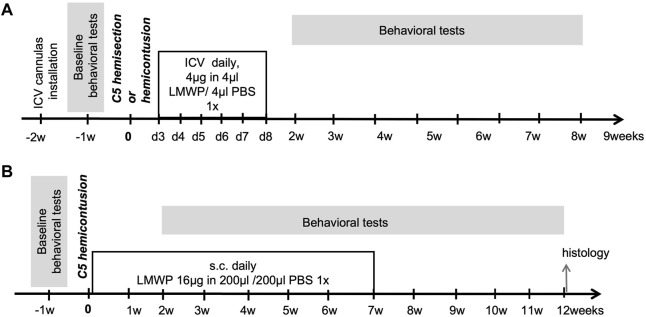
Protocols of *in vivo* experiments. **(A)** Hemisection and hemicontusion experiments using ICV delivered LMWP. **(B)** Hemicontusion experiments using systemic delivery of LMWP.

The effectiveness of LMWP delivered to CNS through ICV cannulas in treatment of spinal cord trauma was tested using the cervical hemicontusion and cervical hemisection models (Figure 1A). The experiments using the hemicontusion and hemisection models were carried out in a similar way (Figure 1A). Before starting the experiments, intracerebroventricular (ICV) cannulas were installed to all animals. After 1 week recovery period the animals were tested in the battery of behavioral tests to assess the baseline level of motor functions. At the age of 12 weeks the animals were split to 3 groups: “sham”, “trauma + PBS” and “trauma + LMWP” considering equal distribution of body weights and the baseline behavioral test parameters between the groups. There were 10 animals per group used in hemisection model, and 15 animals per group in groups with trauma (“trauma + PBS” and “trauma + LMWP”) for hemicontusion model as it results in more severe effect on animals and we expected a higher number of animals to be excluded due to surgery complications. To avoid the housing effect, the animals from one cage were allocated to all three groups. Treatment was started on the third day after spinal cord surgery and continued daily for the following 6 days. Four µl of 1 mg/ml LMWP diluted in sterile PBS or 4 μl of sterile PBS were injected at the rate 1 μl/min to awake animals through ICV cannulas. The sham animals received a PBS injection similarly in the group “trauma + PBS”. For assessment of functional recovery after spinal cord injury and following the treatment we have implemented a battery of behavioral tests that does not require adaptation or learning and could be repeated multiple times to evaluate the progress. Behavioral assessment was started 2 weeks after the SCI and lasted until the week 9 after the surgery.

The effectiveness of systemically delivered LMWP in treatment of spinal cord trauma was tested in the cervical hemicontusion model (Figure 1B). Baseline behavior was assessed 1 week before the trauma surgery, and the results were used for balanced distribution of animals to the 3 experimental groups (“sham”, “trauma + PBS” and “trauma + LMWP”). Each housing cage contained animals from all three groups. Subcutaneous injections to the back area were started on the next day after the trauma and continued daily for 49 days. LMWP was injected subcutaneously at the 16 µg dose in 200 µl of sterile PBS. The groups “sham” and “trauma + PBS” got the same volume of PBS. Behavioral assessment was started 2 weeks after the trauma and finished at the week 12.

##### 2.5.4.2 Intracerebroventricular Cannula Implantation and Drug Delivery

Indwelling cannulas were implanted 10 days before the trauma surgery to the right lateral ventricle. The animals were anesthetized by isoflurane (4%) and fixed in stereotaxic frame by ear bars. After scalp opening and skull cleaning and drying with H_2_O_2_ and 96% C_2_H_5_OH a small hole was pierced by 0.5 mm micro drill burr (19007-05, FST) avoiding the penetration of the dura mater. Self-fabricated indwelling 23 G guide cannula (Kokare et al., 2011) was implanted in sterile conditions unilaterally to the right cerebral ventricle with coordinates −0.2 mm posterior from Bregma, 1 mm lateral to midline and 2.5 mm ventral and fixed by cement to the skull. Guide cannulas were protected with self-fabricated 30 G dum cannula protruded by 0.5 mm. Rimadil (5 mg/kg, Pfizer) was used to control pain after the surgery and the following days if needed. Experimental treatment was started on the third day after spinal cord trauma. Blunt 30 G 3 mm long needle connected by the flexible PE tubing (0.42 mm inner diameter, WPI) to the 10 μl glass syringes (Nanofil IL syringe, 05E, WPI) was used for the intracerebroventricular infusion through the guide cannula to awake animals. Animals got an infusion of 4 ul of LMWP (1 mg/kg) or 1x sterile PBS with the rate 1 ul/min controlled by syringe pump (Microfluidic single programmable Pump 11 Pico Plus Elite, Harvard Apparatus) once a day for 6 consecutive days.

##### 2.5.4.3 C5 Hemicontusion Injury

The method of cervical hemicontusion was adapted with some changes from the work of Streijger and others (Streijger et al., 2013). For trauma induction, the animals were anaesthetized with isoflurane and placed on the warming plate (37°C). After fur shaving and skin disinfection with betadine solution, a longitudinal midline skin incision was made over the lower cervical-upper thoracic part of the spinal cord. Muscle and connective tissue were cut and retracted to expose the cervical area of the spinal cord. We used the prominent T2 lamina as a landmark to find the targeted C5 region. Custom-made spinal stabilizer bars compatible with standard mouse/neonatal rat adapter for stereotaxic frame (WPI) were designed and 3D-printed in polished steel. The pair of stabilizer bars with clamp width 5 mm was positioned below the C5 vertebra transverse processes and tightened. After laminectomy at the C5 vertebral level, the animals were carefully transported to the impactor system using the contusion device Infinite Horizon Impactor (Precision Systems and Instrumentation, Lexington, KY, United States). The impactor tip (diameter 0.75 mm) was targeted to the right side of the spinal cord at the middle point between the central line and the edge. The IH Impactor produced the impact at the force set at 75 kilodynes, the velocity at 100 mm/s and the dwell time 15 s. The reported actual impact force and tissue displacement were marked for every individual animal. Sham surgery identical to the trauma surgery included spinal cord stabilization and laminectomy but not the impact application. After the stabilizer bars were removed, muscles were closed with absorbable Monocryl suture and skin with non-absorbable Ethilon suture.

##### 2.5.4.4 C5 Hemisection Injury

For the trauma exposure the animals were subjected to the surgery procedure similar to the hemicontusion surgery including isoflurane anesthesia, skin and muscle incision and the C5 spinal cord level exposure. After laminectomy the dura was carefully removed and the right cervical spinal hemicord was transected with a 25 G syringe needle from the midline dorsal vessel to the lateral side. The control animals received a sham surgery that included laminectomy while the spinal cord and the dura mater were left intact. Wounds were closed and the animals were allowed to recover and treated after the surgery in a similar way as the animals with the C5 hemicontusion.

##### 2.5.4.5 Pre- and Post-Surgery Care

Fifteen minutes before the surgery start, the animals were injected i.p. with buprenorphine (Temgesic, 0.1 mg/kg) to control the pain and antibiotic (Borgal, 30 mg/kg) to prevent infection. Immediately after the spinal cord surgery, dexamethasone (Rapidexon, 0.2 mg/kg) was injected s.c. to reduce inflammatory swelling and the animals were placed to the warm recovery cage until they regained the ability to move. Rimadil (5 mg/kg, Pfizer) and 9% sodium chloride were given s.c. to control pain and dehydration for the following 3 days until the animals stopped loosing body weight. Home cages were provided with wet food. The body weight and bladder emptying were checked daily during the week after the surgery. A few animals were in severe health condition as the result of the injury operation and did not recover enough to move and eat independently within 4 days and were therefore excluded from the analysis (final amount of animals included to analyses are marked in corresponding figure legends).

##### 2.5.4.6 Behavioral Assessment

###### 2.5.4.6.1 Vertical Screen

The mouse was placed on the brink of a horizontal wire screen (25 × 22 cm, diameter of wires 2 mm spaced at 1 cm) faced to edge. Immediately after that the screen was turned vertically to place the mouse to the upside down position at the lower edge. The time that the animal needed to climb to the upper edge was measured during 1 min. If the animal was not able to climb up or fell down from the screen the maximum time 60 s was marked as the climbing time. The average parameter of three subsequent trials repeated with at least 1 min interval was used for data analysis.

###### 2.5.4.6.2 Cylinder Test of Limb Use Asymmetry

The mouse was placed into the glass cylinder of 13 cm in diameter and 15 cm high. A mirror was positioned at the angle behind the cylinder for better observation. The mouse was videotaped for the first 20 exploratory vertical rearings or for 5 min. The number of rearings in which the mouse used the right (impaired), the left or both limbs for support against the wall was measured. Paw preference was calculated as the ratio of total number of left limb use (left + both) to total number of right (impaired) limb use (right + both). A trial with less than 5 rearings within 5 min was excluded from the analysis.

###### 2.5.4.6.3 Locomotor Activity in Open Field

To evaluate the locomotor activity the animals were released to the corner of a transparent plexiglas chamber (30 cm × 30 cm, MedAssiociates, St.Albans,VT; illumination about 100lx) for 15 min. The travelled distance and the time spent in vertical activity (exploratory rearings) were analyzed with the Activity Monitor v5.1 software (Med Associates, St. Albans, VT).

##### 2.5.4.7 Immunohistochemical Assessment

The samples for immunohistochemistry (IHC) were taken from all animals tested in the hemicontusion study with subcutaneous 7 weeks treatment (Figure 1B) at the end of the experiment at 12 weeks (Figure 1B). The cervical spinal cord segments were sectioned into 40 microns thick transverse sections from tissue blocks (samples embedded into solid OCT compound by freezing with dry ice) by using microtome (Leica CM3050 S). Few spinal cord samples were excluded from the study due to technical problems in spinal cord dissection or sectioning procedures, and the total amount of 11 spinal cords of the “trauma + PBS” and 7 spinal cords of the “trauma + LMWP” group were analyzed. Every fourth section per animal was chosen for IHC neurofilament staining making the section interval 120 microns. The sections were stained with primary antibodies against NF200 (ab8135, Abcam, 1:500) in blocking buffer (1xTBS with 0.5% TX-100 and with 3%BSA) and AlexaFluor 594 (REF A11037, Invitrogen) secondary antibodies at the dilution 1:400 in TBS-T (1xTBS with 0.5% TX-100) on microscope slides. The washings prior to and between the stainings were done with TBS-T, whereas the final wash before mounting with Vectastain mounting medium with DAPI, was done by using reverse osmose water.

The whole spinal cord samples were scanned by using Pannoramic 250 (3D HISTECH) with x20 objective. The sample scans were inspected with CaseViewer (3D HISTECH) and 8 sections per animal around the trauma epicenter were chosen for further analyses. Fiji (NIH) was used to process the sample images (1920 x 1017 pixels) and to make measurements for NF200 positive fibers as area percentage to total area of trauma hemisphere. Prior to measurements, the contrast was enhanced by 0.3%-pixel saturation, the unspecific background subtraction was done with default settings, the remaining unspecific signal was manually traced and removed, and then the image was converted first to 8-bit and then binary image for NF200+ signal measurement. The region of interest was drawn slightly smaller than the trauma hemisphere to avoid staining bordering effect to cause measurement inaccuracies from unspecific staining.

### 2.6 Ethical Approvals

All experimental protocols for neuronal cell culture experiments of the study were approved by ELLA- Animal Experiment Board in Finland (the permission numbers: ESLH-2008-09065/YM-23 and ESAVI/11326/04.10.07/2014). The methods for neuronal cell culture experiments were carried out in accordance with the guidelines of the Animal Experiment Board in Finland. All animal *in vivo* experiments were approved by Country Administrative Board of Southern Finland (ESAVI/13202/04.10.07/2017).

### 2.7 Statistical Analysis

SPSS and GraphPad Prism statistical packages were used for statistical analysis. All data were tested for normality distribution by Shapiro-Wilk test. Latency to climb up in the vertical screen test, paw use ratio in the cylinder test, and the time spent in the center in the open field test displayed deviation from the normal distribution and were analyzed by Kruskal-Wallis test with Dunn’s post-hoc pair-wise comparison. *In vitro* data and TUNEL assay data were analyzed with unpaired t-test or ANOVA followed by Fisher’s LSD test. Repeated measurements of body weight and distance travelled in open field were analyzed using repeated two-way ANOVA with Tukey’s post-hoc test for pair-wise comparison. ROUT method was used for outliers identification. The data are presented as the mean ±SEM.

## 3 Results

### 3.1 Effects of Protamine and its Fragments on Hippocampal Neurons *In Vitro*

**FIGURE 2:**
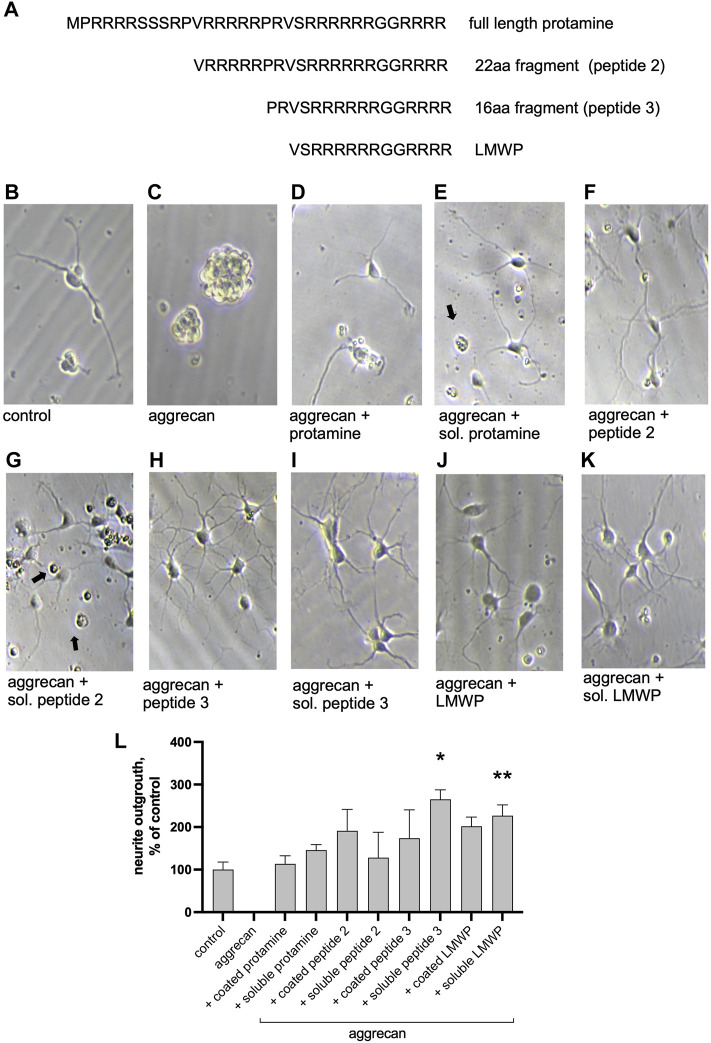
Protamine and its fragments enhance neurite outgrowth on the aggrecan-coated substrate. **(A)** Sequence of the full-length protamine and the tested fragments of 22, 16 and 14 (LMWP) amino acids. **(B–K)** Morphology of hippocampal neurons cultured for 2 days on aggrecan, or on aggrecan with protamine or its peptides (10 μg/ml) that were precoated together with aggrecan or added in the culture medium at the time of cell plating (marked soluble “sol.”). Black arrows point to dead cells. **(L)** Quantification of the effect of protamine and fragments on neurite outgrowth measured from about 70 neurons/replicate and normalized to control level. *p < 0.05, ** *p* < 0.01; ANOVA followed by pairwise comparison with Fisher’s LSD test versus control group; *n* = 3.

We used aggrecan-coated substrates as in our previous studies (Paveliev et al., 2016) to study the effects of protamine and its fragments (Figure 2A) on neurite outgrowth in primary CNS neurons (Figures 2B–K). Cell morphology showed some neurite outgrowth on plain tissue culture plates (Figure 2A), whereas no outgrowth of neurites was observed on the aggrecan-coated plates (Figure 2B). Aggrecan coating together with protamine or its fragments, or adding them in the culture medium after aggrecan coating (soluble protamine or peptide) displayed some neurite outgrowth in all cases. Quantification of the experiment showed that LMWP and the 16 amino acid fragment are more effective in reversing the inhibitory effect of aggrecan than the 22 amino acid fragment or the full-length protamine (Figure 2L). Signs of cell toxicity were observed in neuronal morphology when protamine and the 22 amino acid fragment were tested (black arrows on Figures 2E,G), which may explain their lower efficacy in the neurite outgrowth assay. We then addressed the neurite morphology in more detail (Supplementary Figure S1) and demonstrated that both the number of branches and the length of the longest neurite were similar for the neurons cultured on the uncoated control substrate and on the substrate precoated with aggrecan and protamine (Supplementary Figures S1B,C,E,F). By contrast, LMWP precoated with aggrecan induces a 3-fold increase in the number of branches (Supplementary Figures S1C,F), that is consistent with the increase in the total neurite length (Figure 2 and Supplementary Figures S1A,D), and slightly reduces the length of the longest neurite (Supplementary Figures S1B,E).

**FIGURE 3:**
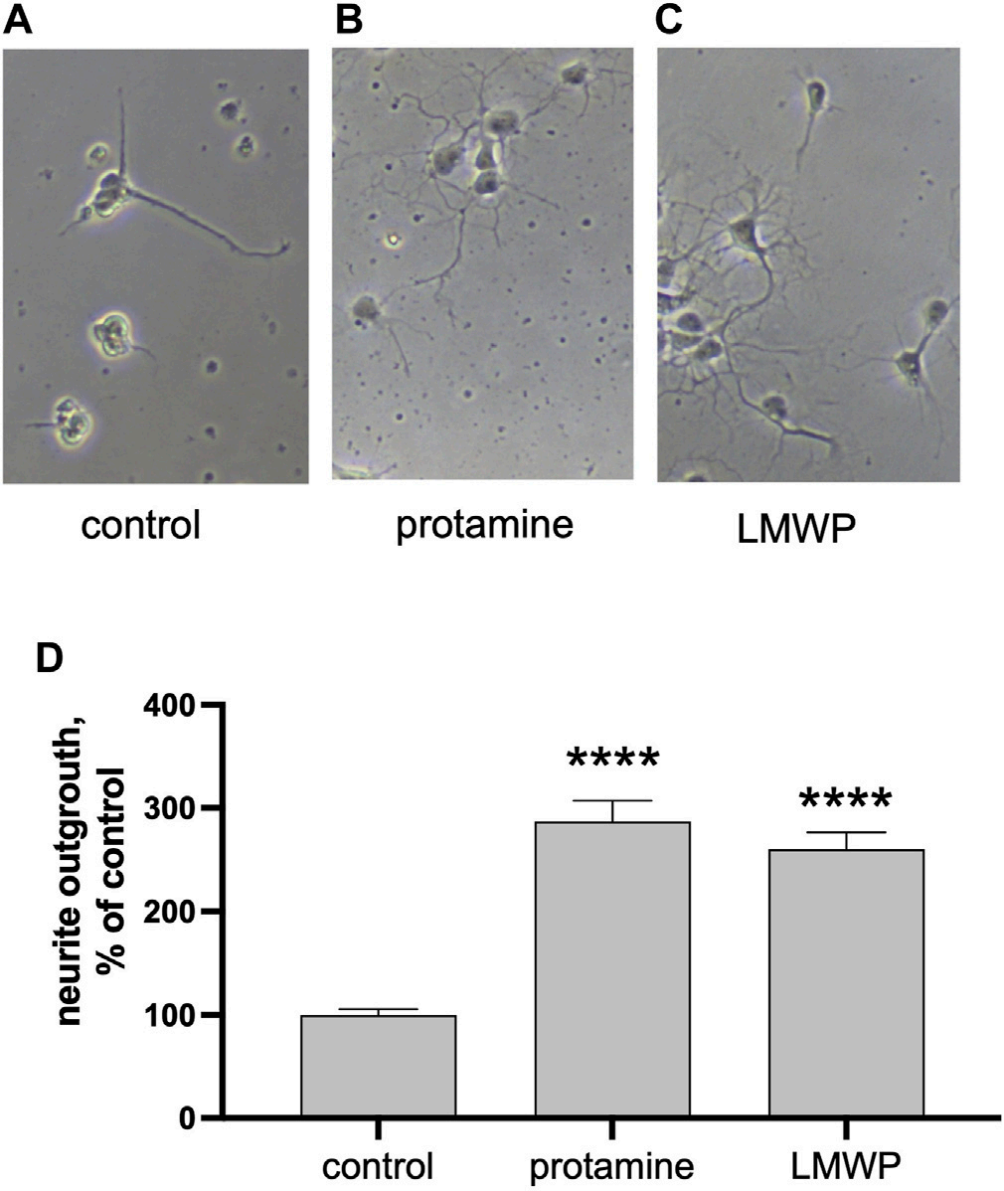
Precoated protamine and LMWP induce neurite outgrowth as compared to control. **(A–C)** Morphology of hippocampal neurons cultured for 2 days on noncoated control plates or plates precoated with 10 μg/ml of protamine or the 14 amino acid fragment LMWP. **(D)** Quantification of the effect of protamine and LMWP on neurite outgrowth measured from about 70 neurons/ replicate and normalized to the control level. **** *p* < 0.0001; ANOVA followed by pairwise comparison with Fisher’s LSD test versus the control group; *n* = 7.

We then asked whether the substrate coating with protamine or LMWP alone is sufficient to increase the neurite growth. Indeed, both protamine and LMWP precoated on the cell culture plate plastic increased the total neurite length as compared to the parallel control cultures plated on the uncoated substrate (Figure 3).

### 3.2 Low-Molecular Weight Protamine has Reduced or No Toxicity *In Vivo*

**FIGURE 4:**
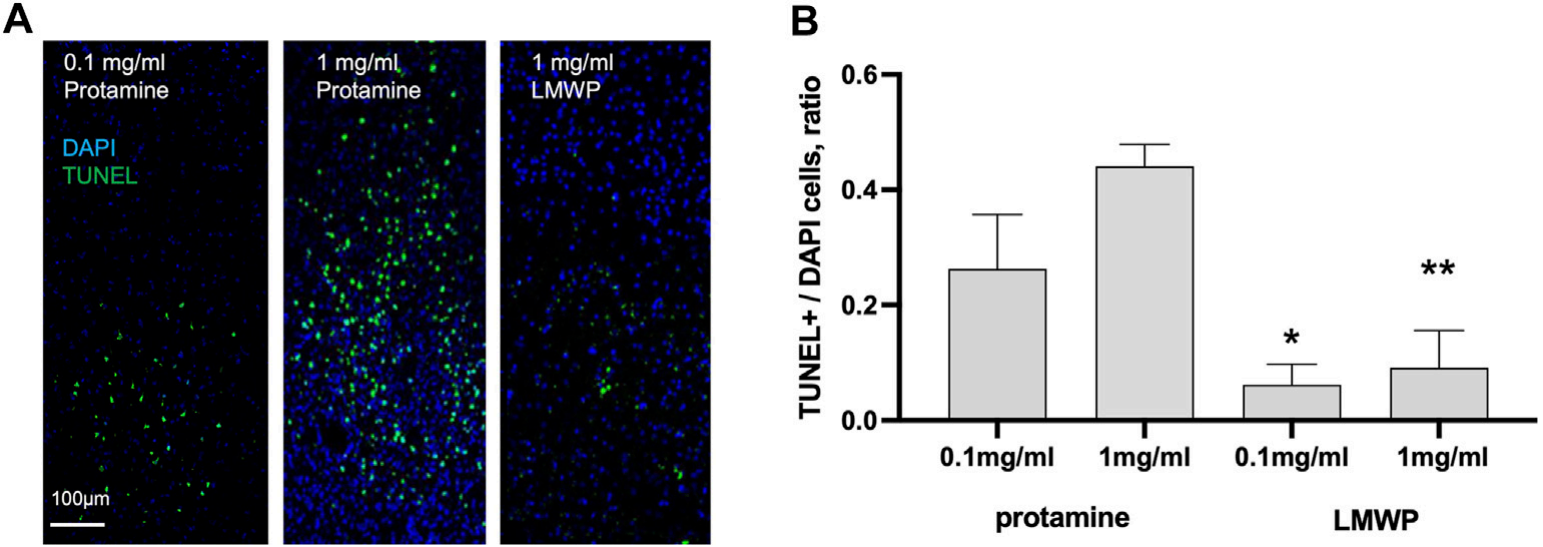
*In vivo* TUNEL apoptosis assay. **(A)** TUNEL staining of the mouse brain sacrificed 3 days after cortical injections of 0.1 or 1 mg/ml (23.6 or 236 µM) protamine in 1.5 µl of PBS compared to 1 mg/ml (532 µM) LMWP in 1.5 µl of PBS. DAPI staining was used for cell nucleus detection. **(B)** Cortical injection of LMWP at the concentration 0.1 or 1 mg/ml (53.2 or 532 µM) reveals significantly less toxicity in TUNEL staining in comparison with protamine injection at the concentrations 0.1 or 1 mg/ml (23.6 or 236 µM). * *p* = 0.0379 compared to protamine at the same mg/ml concentration; ** *p* = 0.0012, ANOVA followed by pairwise comparison with Fisher’s LSD test compared to protamine at the same mg/ml concentration; *n* = 5.

Morphology of CNS neurons cultured in the presence of protamine suggested toxic effects that might be absent when LMWP is used. We therefore decided to test toxicity of protamine and LMWP *in vivo*. While protamine treatment resulted in a high amount of apoptotic cells in brain cortex tissue, TUNEL staining revealed no toxicity in the samples injected even with a high dose of LMWP. Ratio of TUNEL stained cells to total amount of cells labeled with DAPI was significantly lower in cortex tissue treated with LMWP in comparison with tissue treated with protamine at the same mg/ml concentration (Figure 4; *p* = 0.0379 and *p* = 0.0012 for 0.1 mg/ml and 1 mg/ml dose respectively, one-way ANOVA versus corresponding protamine group).

### 3.3 Low-Molecular Weight Protamine-Induced Neurite Outgrowth on Aggrecan Substrate Depends on Chondroitin Sulfate Chains

**FIGURE 5:**
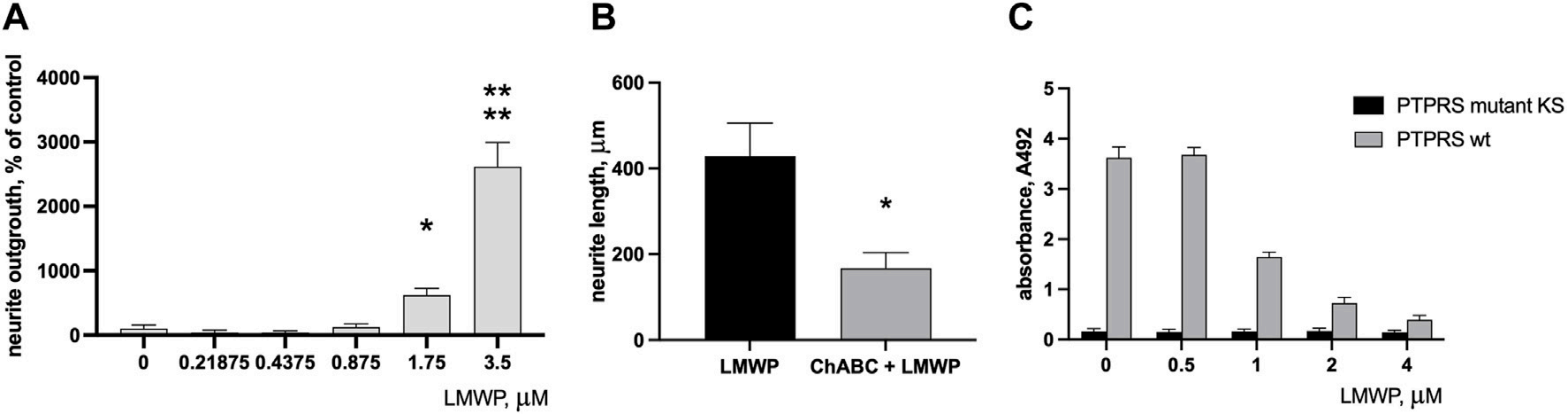
Effect of LMWP on neurite outgrowth on aggrecan substrate and on binding of PTPσ (PTPRS) to aggrecan. **(A)** Neurite outgrowth in cortical neurons on the aggrecan substrate in the presence of increasing concentrations of LMWP added in the culture medium. **(B)** Effect of CS digestion of the aggrecan substrate by chondroitinase ABC (ChABC) on neurite outgrowth on the aggrecan substrate in the presence of 4 µM LMWP. **(C)** Inhibition of PTPσ binding to aggrecan substrate by increasing concentrations of LMWP in the test medium. * *p* < 0.05, **** *p* < 0.0001, unpaired t-test or ANOVA followed by Fisher’s LSD test for multiple comparison; *n* = 3.

LMWP added to the medium stimulated neurite outgrowth in cortical neurons incubated on the aggrecan-coated surface almost 6 times at the concentration 1.75 µM (3.3 μg/ml; *p* = 0.0319, ANOVA followed by Fisher’s LSD test versus concentration 0 μg/ml, *n* = 6) and more than 20 times at the concentration 3.5 µM (Figure 5A) (6.6 μg/ml; *p* < 0.0001, ANOVA followed by Fisher’s LSD test versus concentration 0 μg/ml; *n* = 6). Treatment of the aggrecan-coated plates with chondroitinase ABC that digests the CS chains of aggrecan significantly decreased the neurite outgrowth-promoting effect of LMWP (Figure 5B) (*p* = 0.012, unpaired t-test; *n* = 6). The CS chains of aggrecan are obviously required to bind LMWP to the matrix, and the neurite outgrowth-promoting effect is enhanced by formation of the LMWP-aggrecan complex.

### 3.4 Low-Molecular Weight Protamine Competes With Protein Tyrosine Phosphataseσ for Binding to the Aggrecan Substrate

PTPσ (PTPRS; protein tyrosine phosphatase sigma) has been identified as a transmembrane receptor of neurons that mediates inhibitory effects of aggrecan and related CSPGs on recovery after CNS injuries. We therefore tested whether LMWP interferes in PTPσ binding to aggrecan.

A solid phase binding assay demonstrated that the recombinant ectodomain of PTPσ mutated in a critical lysine residue lacks the ability to bind to CS chains of the aggrecan substrate as expected (Shen et al., 2009). The effect of LMWP mimicked the binding suppression effect seen with the mutated PTPσ. The binding signal of the wild-type PTPσ was progressively decreased by increasing LMWP concentrations in the assay medium (Figure 5C).

### 3.5 Repeated Treatment With Low-Molecular Weight Protamine Through Cerebrospinal Fluid Enhances Functional Recovery in Mouse Model of Cervical Spinal Cord Hemisection

**FIGURE 6:**
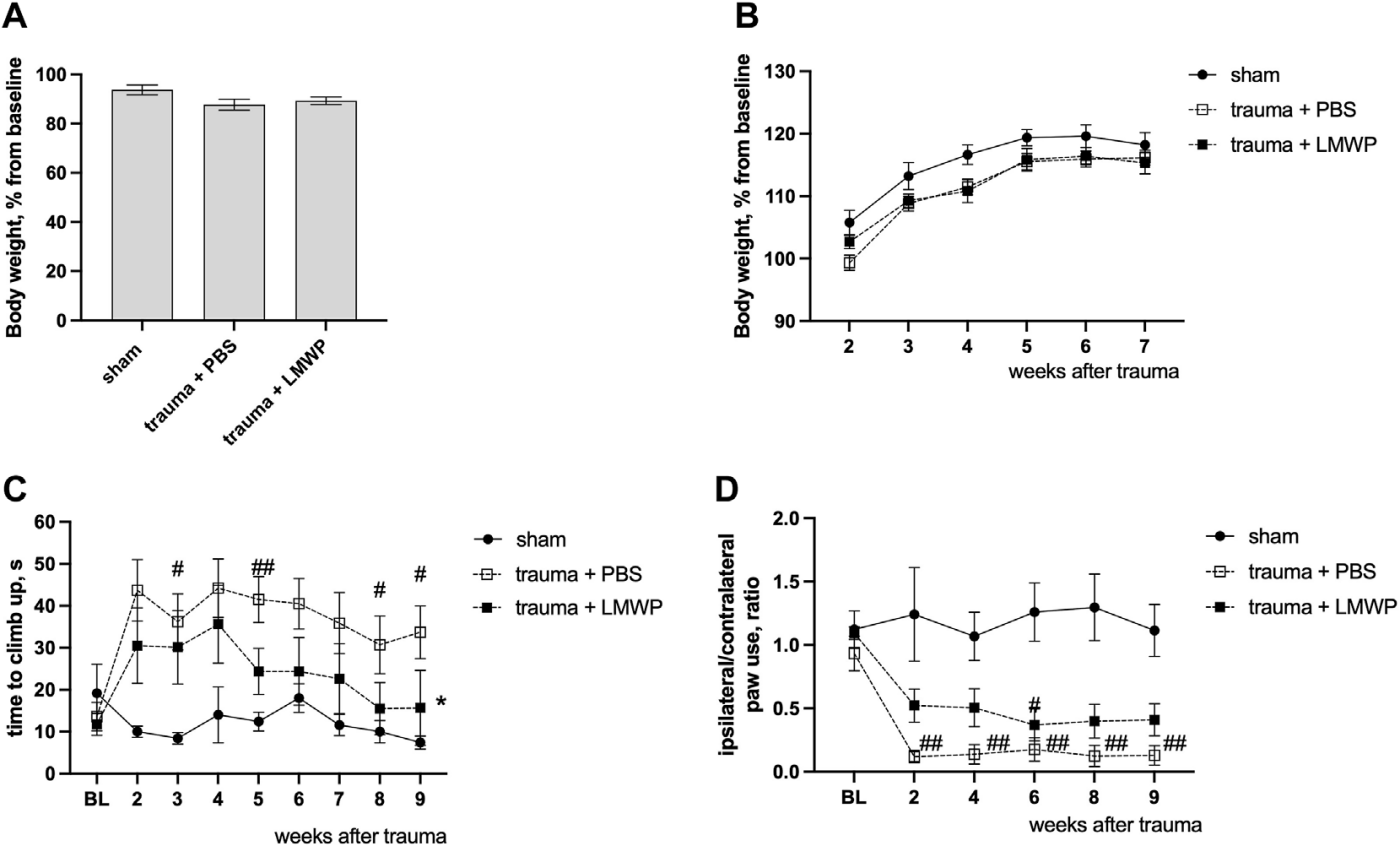
Functional outcomes of LMWP treatment delivered through the intracerebroventricular (ICV) route in the mouse cervical hemisection model. **(A)** The body weight of animals on the day 3 after the trauma before the start of the treatment. **(B)** The change of body weigh during the follow-up behavioral tests. **(C)** The LMWP-treated group demonstrates progressive improvement of climbing function in the vertical screen test. **(D)** The mice treated with LMWP demonstrate reduction of front paw use asymmetry at the weeks 2, 4, 8 and 9 after the trauma. ^#^ *p* < 0.05, ^##^ *p* < 0.01 Kruskal-Wallis test with Dunn’s versus sham group; *p < 0.05 Kruskal-Wallis test with Dunn’s post-hoc pair-wise comparison versus the PBS-treated group; *n* = 5–10.

At the day three after the surgery the animals with spinal cord trauma were equally distributed between treatment groups based on the body weigh values normalized to baseline measurements (Figure 6A) and were treated ICV with PBS or LMWP for six subsequent days. The animals were allowed to recover from the extensive handling stress for a few days, and follow-up behavioral testing to monitor the sensory-motor functions in climbing in vertical screen test and in cylinder test was performed repeatedly from the week 2 to the week 9 after the trauma. Despite the small increase in normalized body weight in the sham animals there were no significant differences between the animal groups during the whole period of follow-up behavioral testing (Figure 6B). The animals with C5 hemisection trauma required significantly more time to reach the top of the vertical screen during the first weeks after the trauma (Figure 6C). During the follow-up period the LMWP group progressively reduced the climbing time while the climbing time for the PBS group stayed significantly higher than that of the sham group until the end of the experiment at the week 9 (week 3 *p* = 0.0223, week 5 *p* = 0.0043, week 8 *p* = 0.0385, week 9 *p* = 0.0147, Kruskal-Wallis test with pair-wise Dunn’s post-hoc comparison versus sham group). The difference in climbing time between the LMWP- and PBS-treated groups reached a statistically significant level at the week 9 after the trauma (*p* = 0.0304, Kruskal-Wallis test with Dunn’s post-hoc pair-wise comparison versus the PBS-treated group).

Moreover, the animals treated with LMWP had less asymmetry in frontpaw use when the injured side was compared to the non-injured side (Figure 6D). The PBS-treated animals demonstrated a significantly lower ratio of ipsilateral paw use during the whole follow-up period in comparison with the sham animals (week 2 *p* = 0.0018, week 4 *p* = 0.0068, week 6 *p* = 0.0026, week 8 *p* = 0.0022, week 9 *p* = 0.0012, Kruskal-Wallis test with pair-wise Dunn’s post-hoc comparison versus sham group). The animals from the LMWP group partially improved the ipsilateral paw use and were not significantly different from the sham animals at the weeks 2 (*p* = 0.4201), 4 (*p* = 0.2766), 8 (*p* = 0.1092) and 9 (*p* = 0.1659, Kruskal-Wallis test with pair-wise Dunn’s post-hoc comparison versus sham group) after the trauma.

### 3.6 Repeated Treatment With Low-Molecular Weight Protamine Through Cerebrospinal Fluid Enhances Functional Recovery in Mouse Model of Cervical Spinal Cord Hemicontusion

**FIGURE 7:**
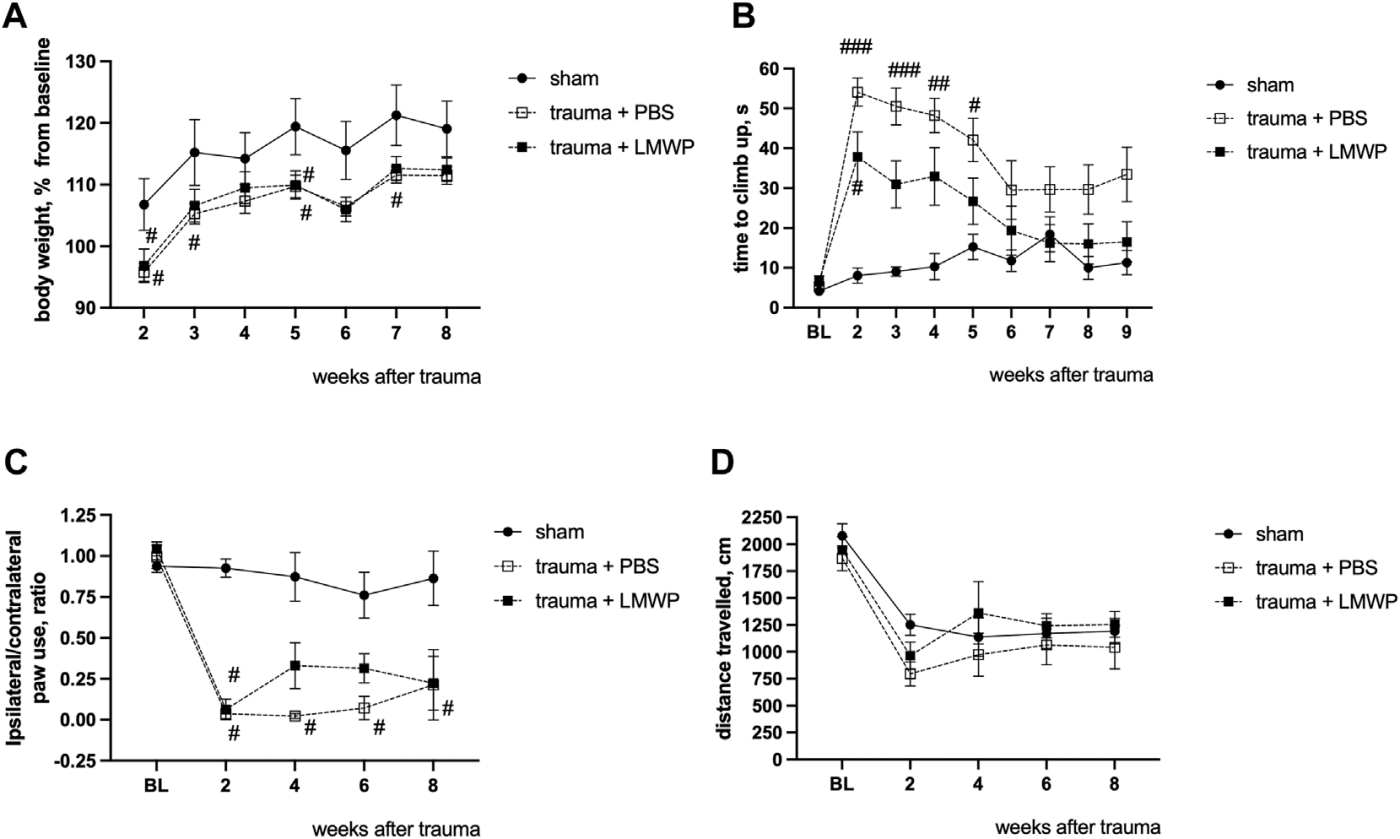
Functional outcomes of ICV-delivered LMWP treatment in the mouse cervical hemicontusion model. **(A)** There are no differences in normalized body weight between the LMWP- and PBS-treated groups during the period of follow-up behavioral testing from the week 2 to the week 8. ^#^ *p* < 0.05, one-way ANOVA with post-hoc Tukey’s pair-wise comparison versus sham group, *n* = 6–11. **(B)** Mice treated with LMWP progressively improve the climbing function in the vertical screen test starting from the week 3 after the trauma induction. **(C)** The LMWP-treated animals demonstrate reduction in front paw use asymmetry starting from the week 4 after the trauma. **(B,C)** # *p* < 0.05, ^###^ *p* < 0.001 Kruskal-Wallis test with Dunn’s post-hoc pair-wise comparison versus the sham group; *n* = 6–11. **(D)** General locomotor activity for 15 min in the open field chamber is quite similar for all animal groups.

To assess the effect of LMWP treatment on sensory-motor functions after the cervical lateral contusion of the spinal cord, a similar set of follow-up tests was carried out as described above in the experiments with the lateral hemisection model. There were no differences in body weight gain between the groups treated with LMWP or PBS during the testing period (Figure 7A). The LMWP-treated mice demonstrated progressive improvement in the vertical climbing test (Figure 7B). Despite of some climbing problems, Kruskal-Wallis test with pair-wise Dunn’s post-hoc comparison did not reveal a significant difference after the second post-trauma week between the sham animals (week 3 *p* = 0.1508, week 4 *p* = 0.1311, week 5 *p* = 0.6967, Kruskal-Wallis test with pair-wise Dunn’s post-hoc comparison versus sham group) and the LMWP-treated group that had undergone hemicontusion, while the vehicle-treated animals used significantly more time to climb up in comparison with the sham animals until the week 5 (week 3 *p* = 0.0006, week 4 *p* = 0.003, week 5 *p* = 0.0186, Kruskal-Wallis test with pair-wise Dunn’s post-hoc comparison versus sham group) (Figure 7B).

Cylinder test demonstrated that all animals with trauma revealed a significant reduction in ability to use the ipsilateral front paw in comparison with the sham-operated mice at the week 2. The PBS-treated animals did not demonstrate any behavioral improvement during the following weeks, while the LMWP-treated group was not significantly different from the sham group on the weeks 4, 6 and 8 after the trauma (Figure 7C).

General locomotor activity was quite similar in all animal groups (Figure 7D). All animals demonstrated a significant reduction in the travelled distance at the week 2 in comparison with the baseline level, while there were no significant differences between the groups during the whole follow-up period.

### 3.7 Repeated Systemic Treatment With Low-Molecular Weight Protamine Using Subcutaneous Injections Enhances Recovery in Mouse Model of Cervical Spinal Cord Hemicontusion

#### 3.7.1 Subcutaneously Injected Fluorescent Low-Molecular Weight Protamine is Detected in the Spinal Cord Parenchyma

**FIGURE 8:**
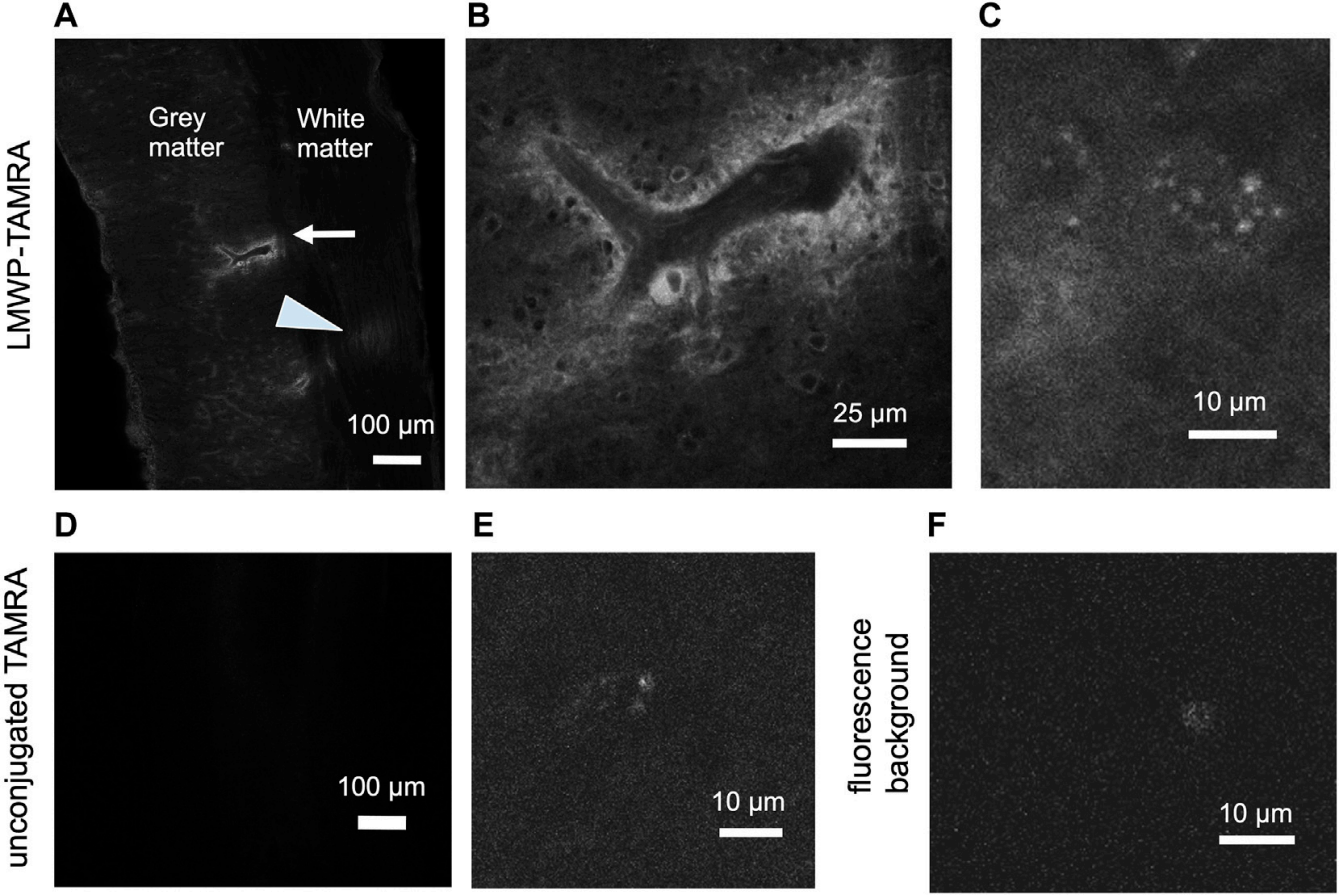
Subcutaneous injection of the fluorescently labelled LMWP results in the fluorescent signal accumulation in the spinal cord parenchyma. **(A)** A longitudinal spinal cord section, cervical region, 2 h after the TAMRA-LMWP injection. Perivascular accumulation of TAMRA-LMWP is marked with an arrow. Fluorescent fiber labeling in white matter is shown with an arrowhead. **(B)** Enlarged view of the perivascular accumulation of TAMRA-LMWP shown in **(A)**. **(C)** Parenchymal accumulation of TAMRA-LMWP. **(D,E)** The cervical spinal cord parenchymal fluorescence 2 h after the unconjugated TAMRA injection used as a control [**(D)** low magnification, **(E)** high magnification]. **(F)** Fluorescence background in the uninjected mouse spinal cord. The scale bar in **(A,D)** is 100 µm, in **(B)** 25 µm, in **(C,E,F)** 10 µm.

Since LMWP is a cell-penetrating peptide and has been reported to pass the blood-brain barrier, we tested whether the fluorescent TAMRA-LMWP can be detected in the spinal cord after subcutaneous injection. Compared to TAMRA or noninjected animals, injection of TAMRA-LMWP resulted in clear fluorescent signal in the spinal cord parenchyma (Figure 8), suggesting that systemic treatment might be possible.

#### 3.7.2 Immunohistochemistry Suggests Enhanced Axonal Regeneration in Spinal Cord Hemicontusion Treated by Systemic Administration of Low-Molecular Weight Protamine

**FIGURE 9:**
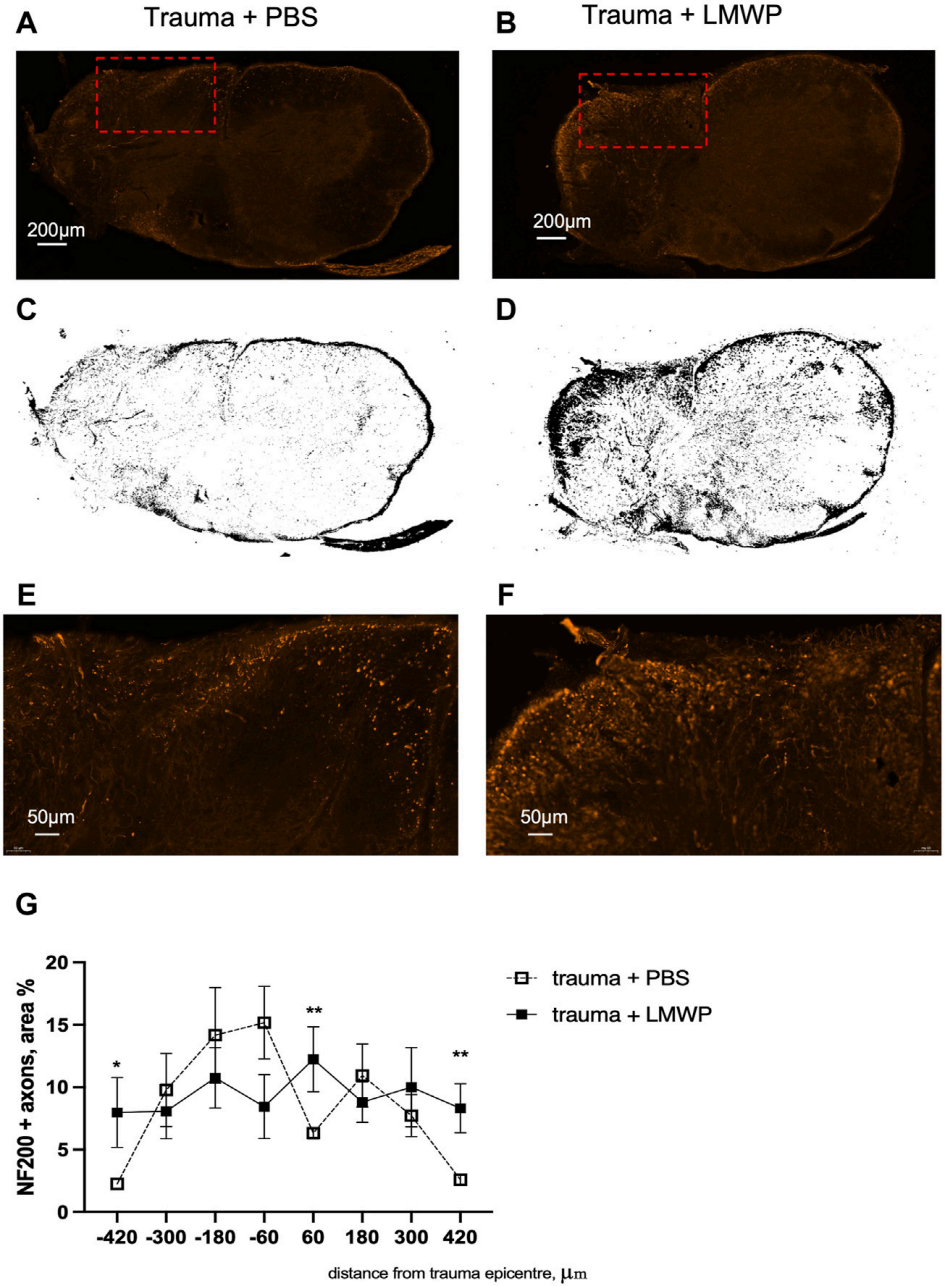
Immunohistohemical staining for NF200+. **(A,B)** Representative images of transverse spinal cord section stained for NF200+ in PBS and LMWP treated mice at the week 12 after hemicontusion trauma induction (scale bar is 200 µm). Red rectangle marks the area enlarged in panel **(E,F)**. **(C,D)** Corresponding threshold images from the sections of PBS- and LMWP-treated animals. **(E,F)** Enlarged view of the NG200+ fibers in PBS and LMWP treated mouse spinal cords (scale bar is 50 µm). **(G)** LMWP treated animals had more NF200+ axons in sections located on the periphery of the trauma epicenter. *p < 0.05, *** *p* < 0.001 Mann-Whitney compared to the same area within trauma + PBS group; *n* = 7–11.

Experiments using systemic LMWP delivery aim at providing information of convenient administration in possible human use. Accordingly, we used systemic subcutaneous delivery in the hemicontusion model that is closer to human injury compared to the hemisection model. There was a statistically significant group difference between the LMWP-treated mice and the control mice in NF200+ fibers (%) in the traced trauma hemisphere sections (Figure 9). LMWP treatment increased the area of the NF200+ signal in the section levels located on the trauma periphery about ±400 µm to caudal and rostral direction from the trauma epicenter (Figures 9E–G; 400 µm to rostral direction *p* = 0.0021, −400 µm to caudal direction *p* = 0.0346; Mann-Whitney). There was also a significant difference of the NF200+ signal area in one section level localized almost in the middle of the trauma epicenter (60 µm to rostral direction, *p* = 0.0064), while there is no similar trend observed in the sections around. The immunohistochemistry results suggest that systemic treatment with LMWP enhances regeneration of neurofilament-positive axons in spinal cord hemicontusion.

#### 3.7.3 Behavioral Outcomes in Hemicontusion Treated by Systemic Administration of Low-Molecular Weight Protamine

**FIGURE 10:**
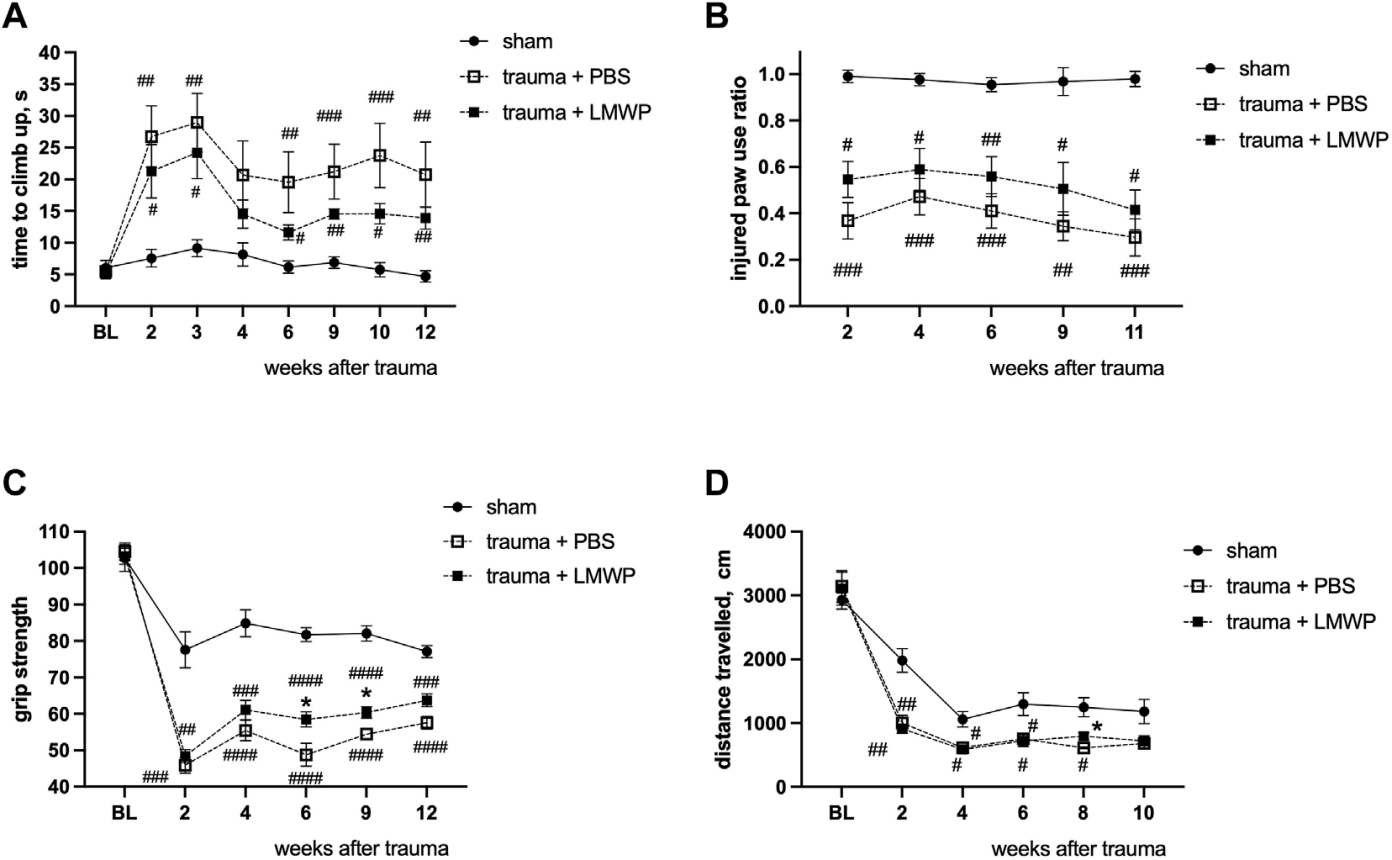
Functional outcome of systemically delivered LMWP in the mouse cervical hemicontusion model. **(A)** Recovery in the climbing test showed a tendency for better recovery in the LMWP group compared to the PBS group. **(B)** Cylinder test for the assessment of asymmetry of front paw use showed a tendency for better improvement of the LMWP group compared to the PBS group although the difference was not statistically significant. **(A,B)** ^#^ *p* < 0.05, ^##^ *p* < 0.01, ^###^ *p* < 0.001, ^####^ *p* < 0.0001 Kruskal-Wallis test with Dunn’s post-hoc pair-wise comparison vs. sham group; *n* = 7–13. **(C)** The LMWP-treated animals demonstrate significant increase of frontpaw grip strength in comparison with the PBS-treated animals on the weeks 6 and 9 after the trauma induction. **(D)** LMWP treatment did not affect general locomotor activity in a 15 min test in open field chamber. **(C,D)** ^#^ *p* < 0.05, ^##^ *p* < 0.01, ^###^ *p* < 0.001, ^####^ *p* < 0.0001 one-way ANOVA with post-hoc Tukey’s pair-wise comparison vs. sham group; *p < 0.05, one-way ANOVA with post-hoc Tukey’s pair-wise comparison versus trauma + PBS group; *n* = 7–13.

In ICV delivery of LMWP, clear improvement was observed in the climbing function and the paw use on the injured side (Figures 6, 7). In the systemic administration, the result was not as clear although a permanent trend of improvement was observed in the same assays (Figures 10A,B). To gain further insight into possible functional improvement, we also analyzed the grip strength (Figure 10C), as we have found it as a useful functional parameter in the hemicontusion model (Kulesskaya et al., 2021).The mice that had received the s.c. injections of LMWP had significantly increased grip strength parameters on the week 6 and the week 12 as well as a trend towards increased grip strength on the week 12 in comparison with PBS-treated animals (week 6 *p* = 0.0449, week 9 *p* = 0.0221, week 12 *p* = 0.0558, one-way ANOVA with Tukey’s multiple comparison test).

The distance travelled in the open field arena was measured to assess general activity of the different mouse groups. As in the ICV delivery experiments (Figure 7D), activity of the different groups did not show statistically significant differences (Figure 10D).

## 4 Discussion

Chondroitin sulfate chains of the CSPGs in the extracellular matrix of the glial scar and the perineuronal nets (Kwok et al., 2011) have been widely studied as inhibitors of plasticity and regeneration. These studies have mainly used chondroitinase ABC treatment to diminish or eliminate the effects of the CS chains (Bradbury et al., 2002; Pizzorusso et al., 2002; Silver and Miller, 2004; Galtrey ad Fawcett, 2007; Garcia-Alias et al., 2009; Sharma et al., 2012). We have recently started an alternative approach to overcome the CS effects by using proteins that bind avidly to the CS chains and might therefore modify the effects of the glial scar and the perineuronal nets.

Our candidate protein to overcome the CS inhibition on neurite outgrowth and regeneration has been HB-GAM (heparin-binding growth-associated molecule; pleiotrophin) that was initially isolated by screening for factors from juvenile rat brain that enhance neurite outgrowth in CNS neurons (Rauvala, 1989; Li et al., 1990; Merenmies and Rauvala, 1990). These studies have shown that HB-GAM indeed overcomes the CS inhibition and enhances neurite outgrowth on CSPG substrates, such as aggrecan and a CSPG mixture isolated from brain (Paveliev et al., 2016). Furthermore, we have recently shown that HB-GAM enhances functional recovery in hemisection and hemicontusion spinal cord injury models in mice (Kulesskaya et al., 2021).

The CS chains even enhance the neurite outgrowth-promoting effect of HB-GAM, and we have ended up in a model according to which the CS chains of the CSPGs display a positive role by concentrating HB-GAM on the matrix and presenting it to neuron surface receptors. According to our model, two polycationic regions separated by a linker are essential to reverse the CS inhibition of neurite outgrowth (Paveliev et al., 2016; Rauvala et al., 2017). To test our hypothesis we decided to search for peptide structures that are similar to HB-GAM and display similar binding properties. Protamine displays similarity to HB-GAM with polycationic amino acid sequences separated by linker regions. HB-GAM binds avidly to heparin (Rauvala, 1989) and in addition to similar carbohydrate epitopes in the CS chains (Milev et al., 1998). Protamine is a well-known heparin-binding protein used globally in cardiopulmonary surgery to neutralize the anticoagulant effect of heparin. Furthermore, protamine also binds to the CS chains of the CSPGs and neutralizes the CSPG-mediated inhibition of oligodendrocyte differentiation (Kuboyama et al., 2017).

Protamine did display activity in overcoming the CSPG inhibition of neurite outgrowth in CNS neurons but proved to be toxic for neuronal cells (Figures 2, 4). We therefore tested protamine fragments that might be nontoxic but would still retain the ability to overcome the CSPG inhibition. A 14 amino acid fragment was found to still display activity in overcoming the CS inhibition on the aggrecan matrix. This fragment is found in the literature as the low-molecular weight protamine (LMWP) with the capability to penetrate through the cell membranes and to carry drug cargoes to tissues including the brain (Xia et al., 2011; He et al., 2014). In our assays in neuronal systems LMWP was found to display little if any toxicity even at high concentrations (Figures 2, 4). This finding is in agreement with published studies where LMWP is described as a non-toxic substitute of protamine (Byun et al., 2000; He et al., 2014; Edwards et al., 2020).

LMWP has two polycationic regions separated by a short linker, a structural feature that appears essential for the action of HB-GAM (Paveliev et al., 2016; Rauvala et al., 2017). When tested *in vitro* using CNS neurons, LMWP clearly mimicked the effect of HB-GAM (Figure 5). As in the case of HB-GAM, treatment of the CSPG substrate with chondroitinase ABC abolished the activity of LMWP to enhance neurite outgrowth. It appears reasonable that the CS chains bind and concentrate LMWP on the substrate and the CS/LMWP complex enhances neurite outgrowth similarly as was found for HB-GAM (Paveliev et al., 2016). According to this model, one polycationic region of LMWP binds to the CS chains of the matrix masking the inhibitory CS effect, and the other polycationic region binds to neuron surface receptors(s) enhancing neurite outgrowth. Masking of cell matrix CSPG effects has been also recently demonstrated for protamine (Kuboyama et al., 2017).

As regarding masking of the inhibitory CS effect, one should consider the possible effect of LMWP on the interaction with PTPσ that has been identified as the transmembrane receptor of neurons mediating the CSPG inhibition on neurite outgrowth (Shen et al., 2009). Furthermore, inhibition of PTPσ signaling by blocking the function of its intracellular domain has been reported to enhance recovery in models of spinal cord injury (Lang et al., 2015) and of multiple sclerosis (Luo et al., 2018). LMWP clearly inhibited PTPσ binding to aggrecan in a similar manner as HB-GAM (Figure 5). Inhibition of PTPσ binding to its CSPG ligand by LMWP is therefore expected to inhibit PTPσ signaling, and thus likely contributes to reversal of CS inhibition of neurite outgrowth.

In the case of HB-GAM we have identified glypican-1 as a neuron surface receptor using unbiased mass spectrometry search of neuron binding sites for the substrate-attached HB-GAM (Paveliev et al., 2016). Glypican-1 is a heparan sulfate proteoglycan linked to the plasma membrane by a glycosyl phosphatidyl inositol (GPI) anchor and it binds to HB-GAM through its heparan sulfate chains. Interaction of glypican-1 with LMWP also appears likely since LMWP binds to heparin/heparan sulfate chains. Another possible cell surface receptor of LMWP is the transmembrane receptor PTPRZ (protein tyrosine phosphatase receptor type Z) that is known to bind protamine (Kuboyama et al., 2017). Further mechanistic studies are currently warranted on the effects of LMWP on cell surface receptors of neurons.

Since LMWP overcomes the CSPG inhibition when assayed with CNS neurons and displays little if any toxicity for neuronal cells, a major question is whether LMWP enhances recovery after CNS traumas. To answer to this question, we have used hemisection and hemicontusion models in mice that were followed by immunohistochemical analysis and by a battery of behavioral tests. Based on neurofilament staining, LMWP enhances axonal regeneration on the trauma area (Figure 9). Further studies are however required to define, whether regeneration of descending axonal tracts or local sprouting of interneurons is affected.

Behavioral analysis using intracerebroventricular (ICV) injections of LMWP revealed a clearly improved performance in both trauma models in the vertical climbing test and in the cylinder test used to measure frontpaw use of the injured side (Figures 6, 7). These differences could be due to changes of general locomotor activity of the mice. However, we did not detect significant changes in general activity of the mouse groups when measured in the open field test. It rather appears that LMWP enhances functional recovery in demanding locomotor functions requiring coordination of the limb movements. Interestingly, reversal of the CSPG inhibition by HB-GAM produces a similar behavioral outcome in that recovery of demanding locomotor functions requiring integration of spatiotemporal signals is enhanced whereas general locomotor activity is not essentially changed (Kulesskaya et al., 2021).

Considering possible use in human therapy, ICV delivery that we used initially in the current study may not be a viable option. LMWP is a cell-penetrating peptide and systemic administration could therefore be effective instead of the ICV administration. We therefore tested whether fluorescent TAMRA-labelled LMWP could be found in the spinal cord after subcutaneous injection and found that this is indeed the case (Figure 8). Furthermore, when assessed by immunohistochemical and behavioral analysis subcutaneously delivered LMWP at a similar dose as was used in the TAMRA-LMWP experiments enhances recovery in the hemicontusion model (Figures 9, 10) that mimics human SCI better than the hemisection model. However, further studies are warranted to define the optimal dose and pharmacokinetics of LMWP when administered through the systemic route.

Cationic arginine-rich peptides (CARPs), such as LMWP, have been recently suggested to represent a novel class of neuroprotective peptides having a multimodal mechanism of action (Meloni et al., 2020). Considering the current results, the neuroprotective role of LMWP in CNS disorders warrants further studies. It is noteworthy that besides being a promising carrier of drug cargoes into tissues including the CNS, LMWP displays prominent beneficial effects of its own in the CNS injuries. Since several CARPs have been reported to enter the CNS, LMWP and related CARPs represent a promising class of compounds to enhance recovery from CNS injuries, such as mechanical traumas and multiple sclerosis.

## Data Availability

The raw data supporting the conclusion of this article will be made available by the authors, without undue reservation.

## Supplementary Material

Supplementary Figure S1 The quantitative characteristics of the neurite morphology in the control group and the groups treated with protamine or LMWP. The distribution plot of the total neurite length **(A)**, the longest neurite length **(B)** and the number of neurites per neuron **(C)** in hippocampal neurons cultured for 2 days on aggrecan, or on aggrecan with protamine or LMWP (10 μg/ml) that were precoated together with aggrecan. Mean values of the total neurite length **(D)**, the longest neurite length **(E)** and the number of neurites per neuron **(F)**. All values were measured from 10 neurons per condition per independent experiment using 3 independent experiments presented in Figure 2. ****p = 0.0001; ANOVA followed by pairwise comparison with Fisher’s LSD test versus control group; n = 3.
