# Supplementary figures and images for: Low-Molecular Weight Protamine Overcomes Chondroitin Sulfate Inhibition of Neural Regeneration
Source: Front Cell Dev Biol. 2022 Apr 25;10:865275. doi: 10.3389/fcell.2022.865275 (PMC9084902; doi:10.3389/fcell.2022.865275)

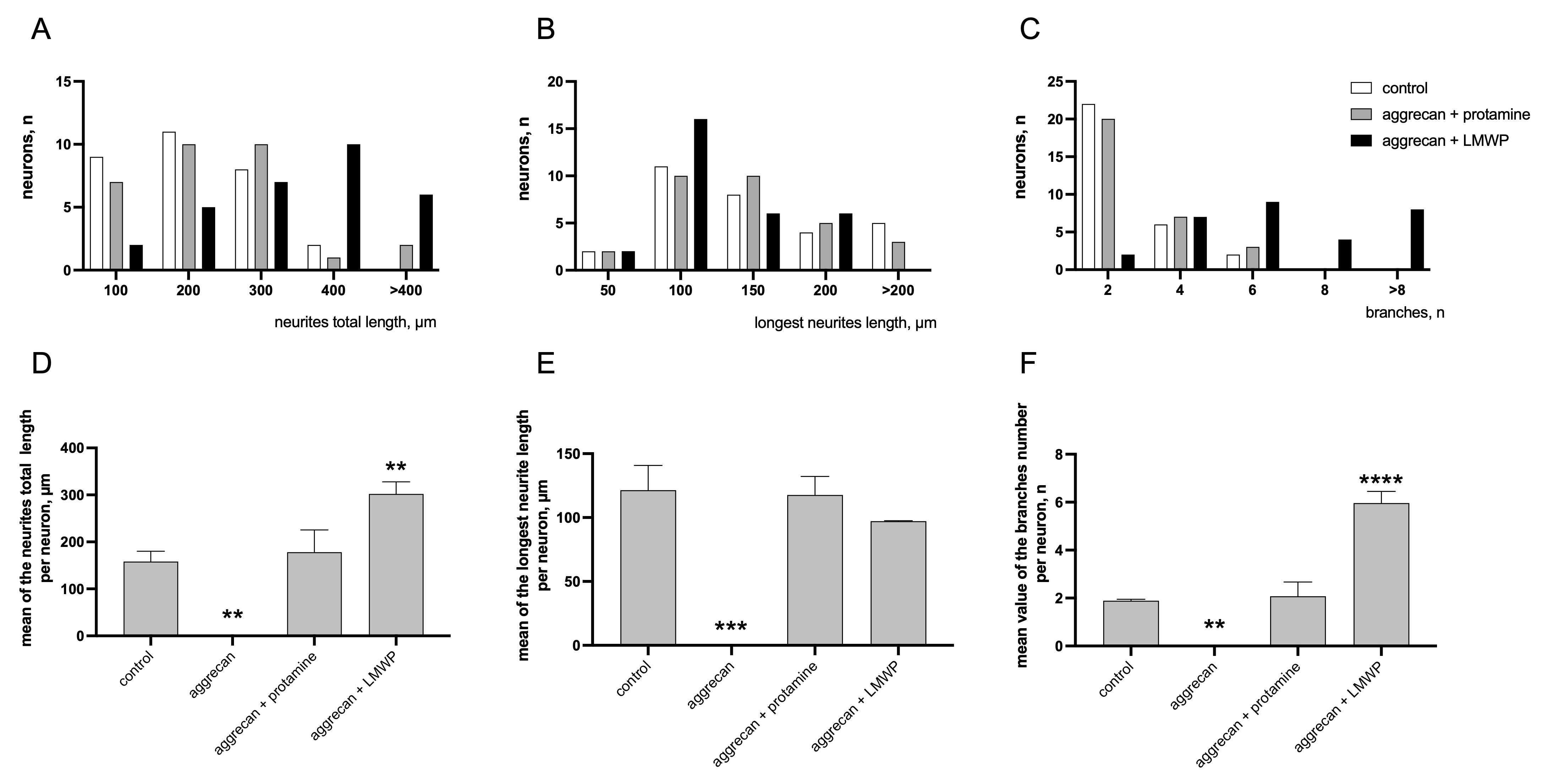

Supplement: Supplementary file 1 [file Image1.JPEG]
